# TREM-1 and TREM-2 as therapeutic targets: clinical challenges and perspectives

**DOI:** 10.3389/fimmu.2024.1498993

**Published:** 2024-12-16

**Authors:** Alexander B. Sigalov

**Affiliations:** SignaBlok, Inc., Shrewsbury, MA, United States

**Keywords:** TREM-1, TREM-2, inflammation, clinical trial failure, multiligand receptors, antagonists & inhibitors, ligand-independent inhibition, drug discovery & development

## Abstract

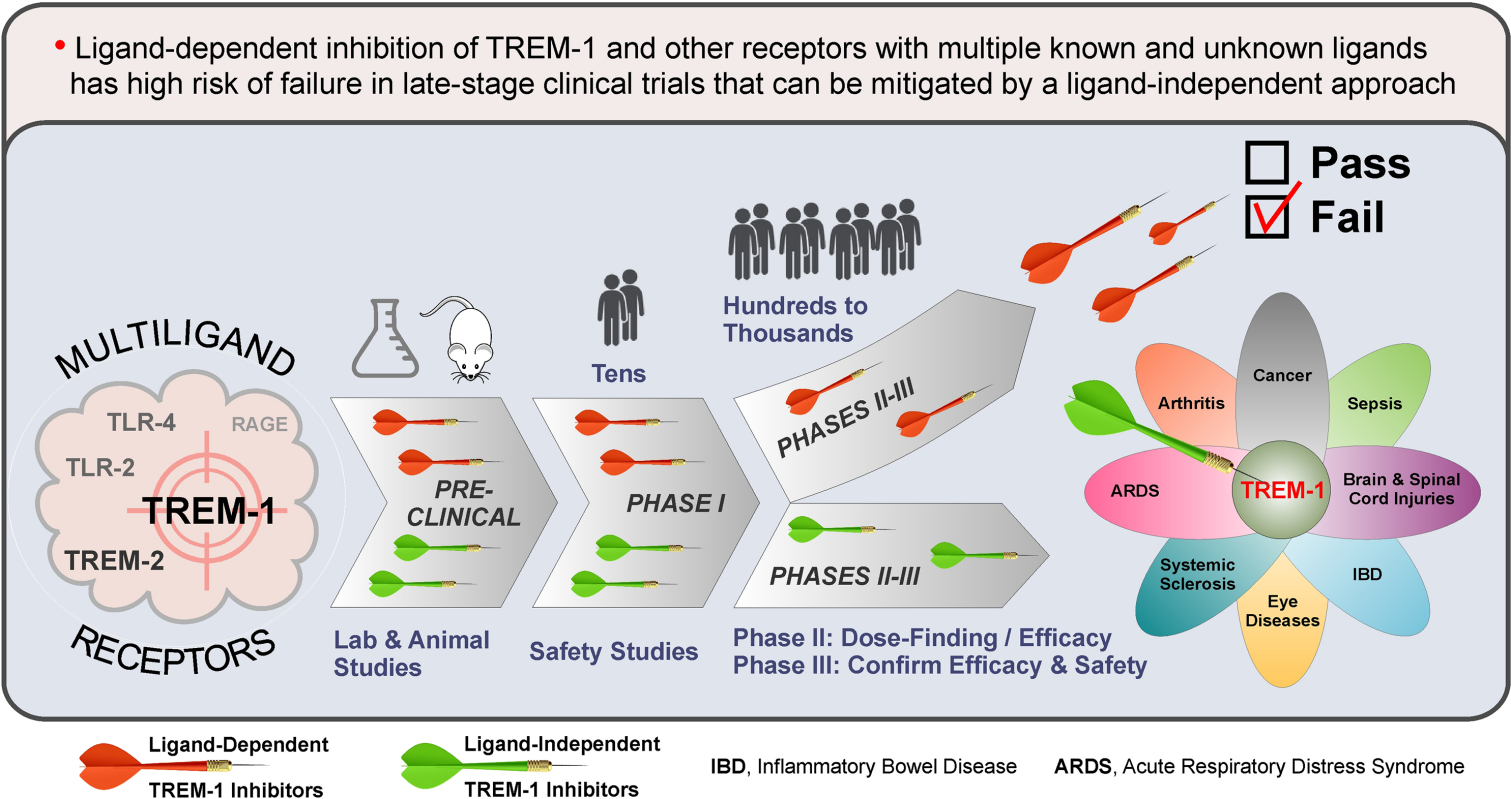
TREM-1 and TREM-2 as Therapeutic Targets: Clinical Challenges and Perspectives.

## 1 Introduction

Triggering receptor expressed on myeloid cells 1 (TREM-1) plays a pivotal role in human health and disease (1–6). Despite promising preclinical data and safety in humans, the first clinical inhibitor of TREM-1 (peptide LR12 or nangibotide) failed to reach significance for the primary endpoint in ASTONISH phase IIb sepsis trial (7, 8). Here, based on the multiplicity of TREM-1 ligands (9–11), I hypothesize that the ligand-dependent mechanism of action of LR12 rather than TREM-1 inhibition per se is the reason of failure. Similar reasoning can explain why an antagonistic monoclonal antibody (mAb) against TREM-2, another multiligand receptor, failed recently in phase Ia/b oncology trials due to lack of efficacy, despite success in animal models (12).

This Opinion focuses on the problems inherent in current, ligand-dependent approaches to pharmacological inhibition of TREM-1, TREM-2 and other multiligand receptors, and suggests solutions to these problems by using ligand-independent strategies that will minimize the risk of failure in the clinic.

## 2 Inhibitors for multiligand receptors

Multiligand receptors are those that specifically interact with multiple ligands (13). Examples are TREM-1 and TREM-2 (5, 14, 15), toll-like receptors (TLR) (16, 17), receptor for advanced glycation end products (RAGE) (18–21), CD36 (22–24) and scavenger receptors (SR) (25–28). Despite numerous preclinical findings supporting a therapeutic value of blocking these receptors, no approved agents are currently available for their specific inhibition.

### 2.1 Poor preclinical to clinical translatability

#### 2.1.1 TREM-1

**Figure 1:**
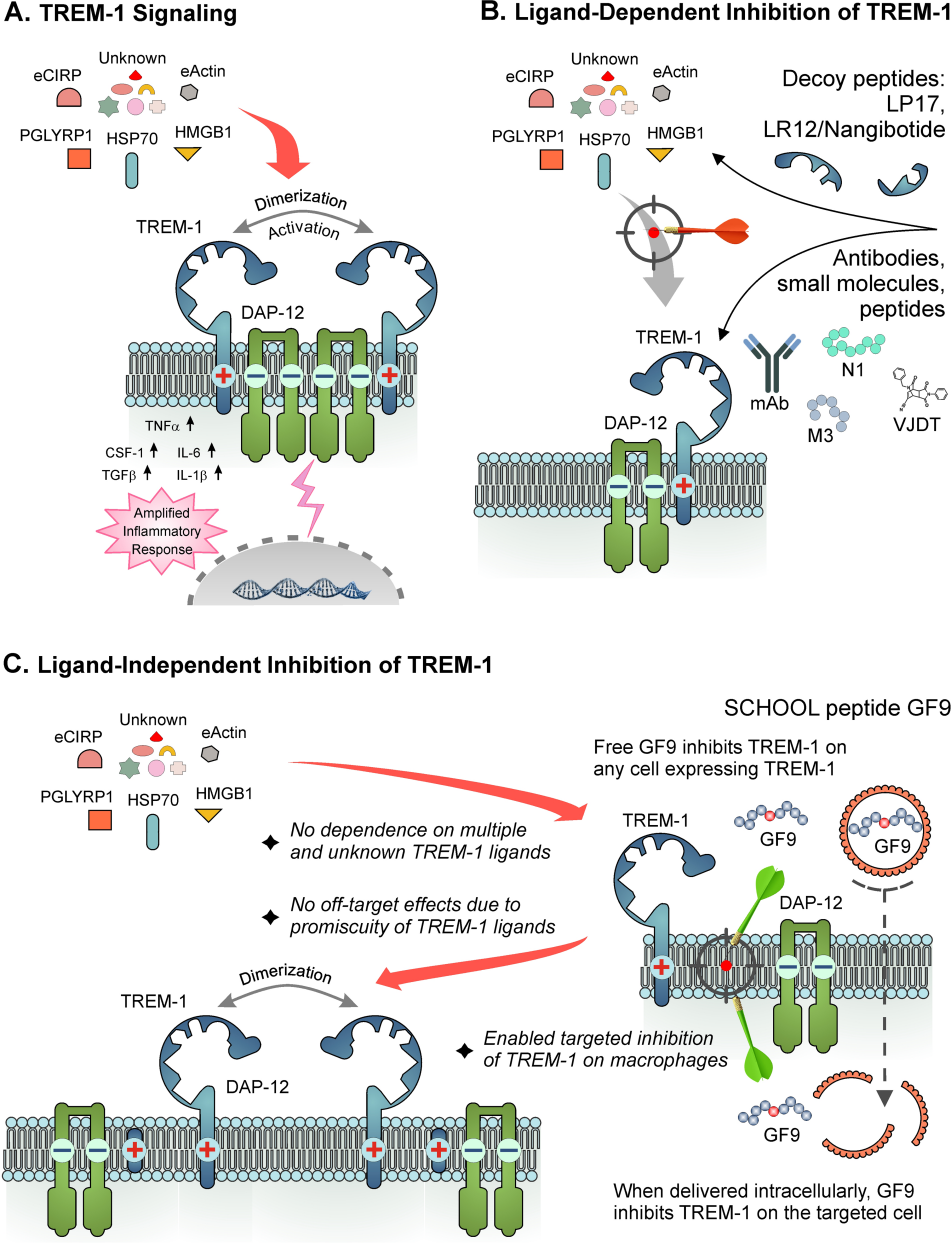
TREM-1: receptor assembly, signaling and mechanisms of ligand-dependent and ligand-independent inhibition **(A)** TREM-1/DAP-12 receptor complex assembly and signaling are depicted. TREM-1 and DAP-12 are bound together by electrostatic interactions between their basic and acidic amino acids in the cell membrane. Binding to the known and unknown natural human ligands of TREM-1 results in dimerization/multimerization of the TREM-1/DAP-12 complex followed by homooligomerization of cytoplasmic DAP-12 domains that triggers the receptor. **(B)** Ligand-dependent inhibitors attempt to block interaction of TREM-1 with its multiple ligands by binding with either the ligands (decoy peptides) or TREM-1 (anti-TREM-1 blocking antibodies, peptides and small molecules). **(C)** Ligand-independent peptide inhibitor GF9 (SCHOOL mechanism-based peptide) disrupts the interactions between TREM-1 and DAP-12 in the cell membrane that upon binding to the ligands, results in dimerization/multimerization of only TREM-1 but not DAP-12, therefore completely blocking the transmembrane signaling. GF9 can reach its site of action in the cell membrane from not only outside but also inside the cell enabling targeted intracellular delivery of GF9 peptide sequence-based TREM-1 inhibitor to certain types of TREM-1-expressing cells. eCIRP, extracellular cold-inducible RNA-binding protein; HMGB1, high mobility group box 1; Hsp70, heat shock protein 70 kDa; PGLYRP1, peptidoglycan recognition protein 1; SCHOOL, signaling chain homooligomerization.

Discovered in 2000 (14), TREM-1, mainly expressed on neutrophils, monocytes, and macrophages, is a key player in the pathogenesis of inflammatory diseases (1–6). TREM-1 is upregulated upon inflammation (5, 29–32) and functions as an inflammation amplifier (5, 14, 33–37) by mediating release of proinflammatory cytokines (5, 38, 39) (**Figure 1A**).

Therapeutic effect of TREM-1 blockade was demonstrated in animal models of sepsis (40, 41), cancer (42–44), acute respiratory distress syndrome (45–47), inflammatory bowel disease (32, 48), rheumatoid arthritis (RA) (49–51), and other inflammation-associated pathologies (52–75).

However, despite promising preclinical efficacy and proven safety in humans (76–82), the first clinical TREM-1 blocker, LR12, failed in the clinic (7, 8). Thus, after more than two decades of intensive research, there are still no approved TREM-1 inhibitors.

#### 2.1.2 TREM-2

First reported in 2001 (83), TREM-2 is expressed on human monocyte-derived dendritic cells, microglia, osteoclasts and tissue macrophage subsets (5, 15). In contrast to TREM-1 that amplifies inflammation (2, 5, 37), TREM-2 acts as a negative (66, 84–88) or positive (51, 89–92) regulator of inflammation.

TREM-2 is overexpressed on tumor-associated macrophages (TAMs) and plays a role in tumorigenesis (5, 93–97). Recent studies (98) resulted in the development of the first anti-TREM-2 antagonistic mAb, PY314, that moved in 2020 into a clinical trial on patients with solid tumors (NCT04691375) (12, 99). However, the limited anti-tumor effect of PY314 observed (12) led to the termination of the trial. Currently, there are no clinical inhibitors of this emerging cancer target.

#### 2.1.3 Other multiligand receptors

*TLRs.* Discovered decades ago (100), TLRs play a role in human health and disease (101) and are emerging targets with wide applicability (102, 103), including the treatment of autoimmune and infectious diseases, and cancer (104, 105). Various TLR-inhibitory therapeutics showed efficacy in preclinical studies but failed at different stages of clinical testing (103, 106) and none have been approved for clinical uses (106).

*RAGE.* First reported in 1992 (107), RAGE is involved in the pathogenesis of inflammation (108–110). Despite decades of interest and research, many RAGE inhibitors that yielded promising preclinical findings failed to translate to humans (108, 109, 111–113). There are no approved therapeutics based on the RAGE antagonists as yet.

*Scavenger Receptors.* CD36 is a multiligand receptor (22, 114, 115) that was first identified in 1977 (116). CD36 is an emerging target in cancer (117, 118) but most of CD36-targeting drug candidates that demonstrated efficacy in preclinical studies failed in humans because of severe adverse events and unsatisfactory efficacy (114). SR-BI, first isolated in 1993 (119), is a multiligand receptor that binds to diverse ligands (120, 121), including lipopolysaccharide (LPS) (122). Despite recognition of SR-BI as an important therapeutic target (123) and promising preclinical findings (123–125), none of the SR-BI inhibitors tested was successful in humans. First described in 1979 (126), SR-A (or CD204) is a multiligand receptor (127) that plays key roles in innate immunity and inflammation (128). Several SR-A inhibitors were reported (129–132), but none is specific for SR-A (132). Thus, there are currently no clinically available inhibitors of CD36, SR-BI and SR-A.

#### 2.1.4 Summary

In summary, despite drug discovery interest and numerous preclinical and clinical studies over several decades, no clinical inhibitors of TREM-1, TREM-2 and other multiligand receptors have been developed to date. I hypothesize that a ligand-dependent mechanism of action of most of the inhibitors tested was and remains a major driver for their failures in humans and that this can be overcome by using a ligand-independent inhibition strategy.

### 2.2 Ligand-dependent mechanism of action: excellence in preclinical, failure in late clinical?

TREM-1 is noncovalently bound in the membrane with its signaling partner, DAP-12, and belongs to a family of multichain immune recognition receptors (MIRRs) (133). According to the Signaling Chain HOmoOLigomerization (SCHOOL) model of MIRR signaling (134, 135), binding of TREM-1 to its ligand(s) results to dimerization/multimerization of the TREM-1/DAP-12 complex followed by homooligomerization of DAP-12 that triggers the receptor (**Figure 1A**).

Since 2015 when the first ligand for TREM-1, PGLYRP1, was identified (9), at least, four other potential ligands have been reported: actin, eCIRP, HMGB1, and Hsp70 (10, 11). It is likely that still more remain unidentified and uncharacterized at present.

Most of current strategies for inhibition of TREM-1 use ligand-dependent inhibitors such as decoy receptor/peptides or anti-TREM-1 antagonistic mAbs (**Figure 1B**). Mechanistically, they attempt to block the interactions of TREM-1 with its multiple known and unknown ligands by binding either to ligands (e.g., decoy peptides LP17 and LR12 (81, 136) or to TREM-1 (e.g., an anti-TREM-1 blocking mAB (48); human eCIRP-derived peptide M3 (70, 73, 137, 138); PGLYRP1-derived peptide N1 (139, 140) and small molecule VJDT (141, 142). Despite the efficacy of these agents in animal models of sepsis (41) and other inflammatory diseases (48, 55, 67, 70, 74, 137–139, 141, 143), only one of them, LR12, has entered clinical trials but failed in phase IIb (7, 8, 41).

Similarly, a humanized anti-TREM-2 mAb PY314 exhibited potent anti-tumor activity in mouse cancer models (144), but was ineffective in the clinical setting (12). Notably, the majority of inhibitors for other multiligand receptors that succeeded in animals but failed in humans (106, 109, 113, 114, 145) were also ligand-dependent.

My hypothesis is that traditional approaches aiming to develop drugs that interfere with the interactions of TREM-1, TREM-2 and other multiligand receptors with their multiple known and unknown ligands all bear a high risk of failure not only in preclinical but also in clinical efficacy testing. This is illustrated on the example of TREM-1 and based on the following major considerations.

First of all, in preclinical testing, potential TREM-1 inhibitors are not and practically cannot be evaluated for their efficacy in blocking interactions between TREM-1 and all its multiple ligands of human origin. Thus, a clinical efficacy trial is the first setting where TREM-1 inhibitory drug candidates compete with known and unknown human ligands or TREM-1 by interaction either with ligands (decoy receptor/peptides) or receptor (anti-TREM-1 blocking antibodies) (**Figure 1B**). The diverse roles played by different ligands of TREM-1 in the pathogenesis of inflammatory diseases and the likely existence of not yet discovered ligands of TREM-1 add more uncertainty to the outcome of clinical efficacy trials. In addition, as demonstrated in sepsis, the expression levels and timing of different TREM-1 ligands may vary depending on the stage of sepsis and type of infection (146–148), further increasing risk of failure of ligand-dependent TREM-1 inhibitors.

Second, decoy peptide-based inhibitors (e.g., LR12) may have off-target effects due to ligand promiscuity. For example, the biological function of HMGB1 is mediated by not only TREM-1 but also other receptors such as TLRs and RAGE, which are expressed on different cells (149). Thus, by binding to HMGB1, LR12 can affect other signaling pathways.

Third, ligand-dependent inhibitors of TREM-1 that bind to ligands (LP17 and LR12) or receptor (anti-TREM-1 blocking mAbs, peptides M3 and N1, and a small molecule VJDT) (**Figure 1B**), all are “pan-TREM-1” inhibitors, i.e., they inhibit TREM-1 on all TREM-1-expressing cells. However, these cells may play different roles in the pathogenesis of infectious and non-infectious inflammatory diseases. For example, in mice with *Pseudomonas aeruginosa*-induced pneumonia, TREM-1/3 deficiency in neutrophils causes increased mortality due to the failure to clear lung bacteria (150). Increased bacterial growth and dissemination and decreased survival were also observed in TREM-1/3-deficient mice with *Klebsiella*-derived pneumosepsis (151) and pneumococcal pneumonia (152).

In summary, for ligand-dependent inhibitors of TREM-1, TREM-2 and other multiligand receptors, promising preclinical and safety studies may not translate to clinical efficacy. This risk can be mitigated by using the inhibitors that employ ligand-independent mechanisms of action.

### 2.3 Ligand-independent mechanism of action: minimizing risk of drug failure

The interactions in the membrane between ligand-recognizing and signal-transducing MIRR subunits can be targeted by short synthetic peptides with receptor-specific sequences (153–155). TREM-1 inhibitory peptide GF9, designed to disrupt the interactions between TREM-1 and DAP-12, thus preventing formation of DAP-12 homooligomers (**Figure 1C**), ameliorates various inflammation-associated diseases in animal models (51, 65, 71, 153, 156–158). Peptide IA9, designed for ligand-independent inhibition of TREM-2, suppresses joint inflammation and damage in experimental RA (51).

In addition to their ligand-independent mechanism of action, another chief advantage of GF9 and IA9 is that their site of action in the membrane enables the development of targeted therapies. As illustrated for TREM-1 (**Figure 1C**), when systemically administered, the GF9 peptide inserts into the membrane of any cell and inhibits TREM-1 wherever it is expressed, acting thus as a pan-TREM-1 inhibitor. When delivered intracellularly to TREM-1-expressing cells of interest (e.g., macrophages including TAMs), GF9 exerts its therapeutic action by inserting into the membrane from inside the cell. This key feature allowed to develop macrophage-targeted formulations of GF9 sequence-based inhibitors effective in suppressing TREM-1-mediated macrophage activation and ameliorating inflammatory diseases in several animal models (51, 71, 153). Following a similar strategy, a macrophage-targeted formulation of IA9 sequence-based TREM-2 inhibitor was designed and demonstrated to ameliorate arthritis in mice (51).

Thus, the use of ligand-independent inhibitors of TREM-1 and TREM-2 not only addresses the multiplicity and promiscuity of their multiple known and unknown ligands, but also enables targeted delivery of these inhibitors to block TREM-1 and TREM-2 on selected cell types. This addresses the potential for different cell types playing different roles in the pathogenesis of many diseases.

Ligand-independent inhibition strategies have been also considered for other multiligand receptors. TLR2 inhibition via blockage of TLR2 dimerization has been recently reported (159). An anti-human TLR4 mAb, NI-0101, that blocks TLR4 dimerization (160) showed efficacy in healthy volunteers receiving LPS (161). Several small molecules that inhibit RAGE by blocking the interaction of the RAGE cytoplasmic domain with DIAPH1 are currently in development (111, 162, 163).

## 3 Conclusion and perspectives

Despite growing interest in therapeutic targeting TREM-1 and TREM-2 over the past two decades (2, 5, 15, 40, 42, 43, 66, 95, 96, 98, 164–168), promising preclinical findings for the first clinical inhibitors of TREM-1 (LR12) and TREM-2 (an anti-TREM-2 antibody PY314) did not translate into clinical efficacy in sepsis (7, 8) and oncology (12) patients, respectively.

To my knowledge, this Opinion is the first attempt to analyze poor preclinical to clinical translatability of inhibitors for TREM-1 and TREM-2 from the angle of multiplicity of their known and unknown ligands and extend this analysis to other multiligand receptors. In summary, deploying a ligand-independent mechanism of action for the pharmacological inhibition of TREM-1 and TREM-2 could bridge the efficacy gap between preclinical and clinical testing observed for ligand-dependent inhibitors as well as mitigate the risk of drug failure. Importantly, the existing preclinical development path may not reveal any difference between ligand-dependent and ligand-independent inhibitors of TREM-1 and TREM-2 since in animal models, these inhibitors do not compete with various TREM-1 and TREM-2 ligands of human origin.

In conclusion, ligand-independent inhibition is a promising alternative strategy to target TREM-1 and TREM-2 in human disease. By addressing the multiplicity and promiscuity of TREM-1 and TREM-2 ligands as well as the differential roles played by these receptors expressed on different cells, this approach inherently mitigates the risk of failure in the clinic, which can save time and resources and substantially increase the odds of success in developing of novel drugs targeting the TREM-1 and TREM-2 signaling pathways.
